# A New Graph-Based Molecular Descriptor Using the Canonical Representation of the Molecule

**DOI:** 10.1155/2014/286974

**Published:** 2014-07-22

**Authors:** Hamza Hentabli, Faisal Saeed, Ammar Abdo, Naomie Salim

**Affiliations:** ^1^Faculty of Computing, University Technology of Malaysia, 81310 Skudai, Johor, Malaysia; ^2^Information Technology Department, Sanhan Community College, Sana'a, Yemen; ^3^Computer Science Department, Hodeidah University, Hodeidah, Yemen

## Abstract

Molecular similarity is a pervasive concept in drug design. The basic idea underlying molecular similarity is the similar property principle, which states that structurally similar molecules will exhibit similar physicochemical and biological properties. In this paper, a new graph-based molecular descriptor (GBMD) is introduced. The GBMD is a new method of obtaining a rough description of 2D molecular structure in textual form based on the canonical representations of the molecule outline shape and it allows rigorous structure specification using small and natural grammars. Simulated virtual screening experiments with the MDDR database show clearly the superiority of the graph-based descriptor compared to many standard descriptors (ALOGP, MACCS, EPFP4, CDKFP, PCFP, and SMILE) using the Tanimoto coefficient (TAN) and the basic local alignment search tool (BLAST) when searches were carried.

## 1. Background

The foundation of a chemical information system is the ability to represent molecules in a computer and to compare a molecule's structure with another. Molecular comparison has been used in the early chemical information systems, for example, structure and substructure searching [1, 2]. Structure searching involves searching a chemical database for a particular query structure to retrieve all molecules with an exact match to the query structure, whereas substructure searching retrieves all molecules that contain the query structure as a subgraph [3, 4]. The equivalence similarity between two structures can be achieved by using a graph and subgraph isomorphism algorithms. Isomorphism algorithms are time consuming because it is a combinatorial problem. Various isomorphism algorithms have been developed for efficient performance but they are too slow for large chemical databases. However, structure and substructure searching were later complemented by another searching mechanism called similarity searching [5].

Similarity searching methods may be the simplest tools for ligand based virtual screening. The basic idea underlying similarity searching is the similar property principle, which states that structurally similar molecules will exhibit similar physicochemical and biological properties [6]. Over the years, many ways of measuring the structural similarity of molecules have been introduced [7–9]. The 2D similarity methods can be divided into two classes: the first class is the graph-based similarity methods and the second one is the fingerprint-based similarity methods. The graph-based similarity methods directly compare the molecular structures with each other and identify the similar (or common) substructures. These methods relate parts of one molecule to parts of the other molecule, and they generate a mapping or alignment between molecules. Maximum common subgraph method (MCS) is an example of the graph-based similarity methods. Another example of the graph-based similarity methods is the feature trees. The feature trees were introduced by Rarey and Dixon [8], which are the most abstract way of representing a molecule by means of a graph. A feature tree represents hydrophobic fragments and functional groups of the molecule and the way these groups are linked together. Each node in the tree is labelled with a set of features representing chemical properties of the part of the molecule corresponding to the node. The comparison of feature trees is based on matching subtrees of two feature trees onto each other. Feature trees allow similarity searching to be performed against large database, when combined with a fast mapping algorithm [9]. However, the most common similarity approaches use molecules characterized by fingerprints that encode the presence of fragments features in a molecule. The similarity between two molecules is then computed using the number of substructural fragments that is common to a pair of structures and a simple association coefficient.

The shape similarity between two molecules can be determined by comparing the shapes of those molecules; find the overlap volume between them and then use similarity measure (e.g., Tanimoto) to calculate the similarity between the molecules. However, most of the works in shape-based similarity approaches depended on the 3D molecular shape. The shape comparison program rapid overlay of chemical structures (ROCS) [10] is used to perceive similarity between molecules based on their 3D shape. The objective of this approach is to find molecules with similar bioactivity to a target molecule but with different chemotypes, that is, scaffold hopping. However, a disadvantage of 3D similarity methods is that the conformational properties of the molecules should be considered and therefore these methods are more computationally intensive than methods based on 2D structure representation. The complexity increases considerably if conformational flexibility is taken into account. There are many 2D structure representations in a numerical form integer or real. The simplest 2D descriptors are based on simple counts of features such as hydrogen donors, hydrogen bond acceptors, ring systems (such as aromatic rings), and rotatable bonds, whereas the complex 2D descriptors are computed from complex mathematical equations such as 2D fingerprints and topological indices. Topological indices are integer or real value numbers (single value) that represent the constitution of the molecules and can be calculated from the 2D graph representation of molecules and may contain additional property information about the molecule [11]. They characterize molecular structures according to their size, degree of branching, and overall shape where the structural diagram of molecules is considered as a mathematical graph but not the contour of molecule shape.

One of the representation types of chemical structures is the line notations, which encodes the connection table and (usually) the stereochemistry of a molecule as a line of text. They are widely used for storing, representing, communicating, and checking the identity of chemical structures. Their popularity derives from one or more of the following: they encode the chemical structure in a compact form; they may be human-readable and/or human-writable; they are easily entered into software (e.g., by copying and pasting into a text entry box on a website); they may be canonical (i.e., provide a unique representation for a particular molecule), in which case they may easily be used to check identity, search databases, or even search the web. In [12, 13], we introduced a new language for writing descriptors of outline shape of molecules (LWDOSM) and LINGO for descriptors of outline shape of molecules (LINGO-DOSM) that were inspired by research in information retrieval on the use of contour-based shape descriptor for image retrieval systems. The LWDOSM is a new method to obtain a rough description of the 2D molecular structure from its outline shape and allows rigorous structure specification by the use of a very small and natural grammar. Typically, there may be many different ways to construct the connection table and the LWDOSM string for a given molecule. In a connection table, one may choose different ways to number the atoms and coordinate each atom; LWDOSM string may be written starting from the top left atom and by following a different sequence through the molecule to get final LWDOSM. For example, a number of equally valid LWDOSM can be written for a molecule. For example, C–C–O–C, C–O–C–C, O–C–C–C, and C–C–C–O all specify the same structure of ethanol C_2_H_6_O. This problem could be tackled by renumbering one of the connection tables in all possible ways and testing for identity. However this is computationally infeasible since there are N different ways of numbering a connection table consisting of N atoms. Hence, in order to deal with this situation it is usual to generate a canonical representation of the chemical structure. A canonical representation is a unique ordering of the atoms for a given molecular graph.

In this paper, an algorithm has been developed to ensure that the same representation is generated for a molecule regardless of the order of atoms in the structure. This graph-based descriptor for molecule (GBMD) provides unique representation for each structure and depends on the canonicalization algorithm. A well-known method for determining a canonical order of the atoms is the Morgan algorithm [14] which is used in this algorithm.

## 2. Methods

The new descriptor (GBMD) is a textual descriptor using printable characters for representing molecules. In this work, the proposed method uses a connection table to extract the information needed to represent the molecule. The GBMD is a true language, albeit with a simple vocabulary (atom and bond symbols) and only a few grammar rules. However, part of the GBMD power is that it is highly sensitive to molecular structure changes. This is because each atom and bond is recorded more than once (back and forth).

In this work, the graph denotes the 2D molecular structure. Here, only the labeled molecular graph (i.e., atoms and bonds) and all possible paths between every atom pair are taken into account. A correspondent shape to a 2D molecule structure is generally composed of a main region (representing the outline shape) and one or many internal regions (representing areas inside rings) obtained after visiting all the atoms in the connection table of a molecule. The process of generating the shape descriptor of any molecule involves few steps as follows.

**Step 1 .**

**Figure 1:**
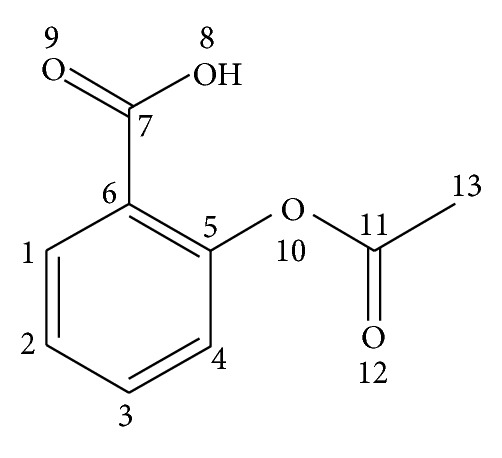
Aspirin molecule structure.

Define graph G = (V, E) to represent the molecule M. Each vertex and edge of G represents atom and bond, respectively, in molecule M. The vertices and edges are labeled with the corresponding kind of atoms and bonds, respectively (see Figure 1).

**Step 2 .**

**Figure 2:**
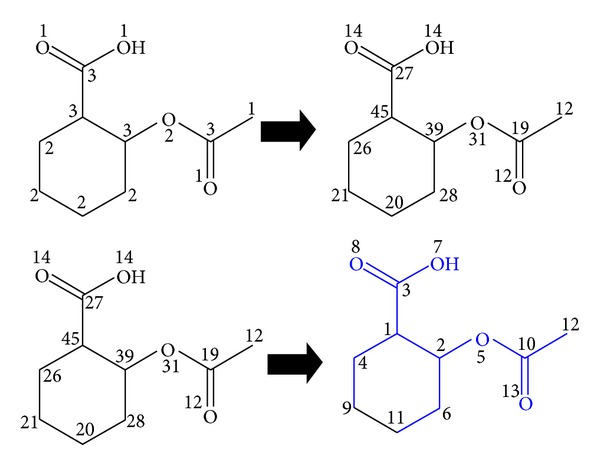
Construct atomic connectivity values in GBMD using Morgan algorithm.

Construct atomic connectivity values in GBMD using Morgan algorithm as shown in Figure 2.

**Figure 3:**
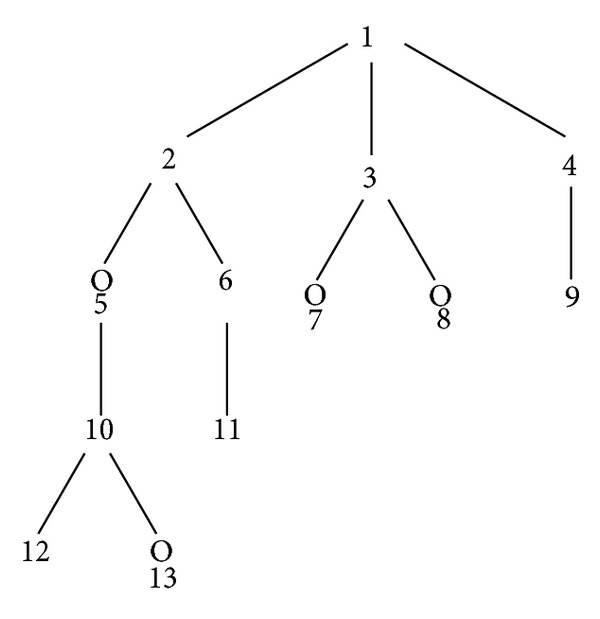
Graphical representation of tree graph extracted from aspirin molecule graph.

Once vertices' numbering in graph G is completed, a tree graph T is constructed. T is a subgraph from graph G that contains all the nodes (but not necessarily all the edges as highlighted in Figure 2 using the blue color) of a graph G.  Figure 3 shows T graph extracted from graph G represents the aspirin molecule.

**Step 3 .**

**Figure 4:**
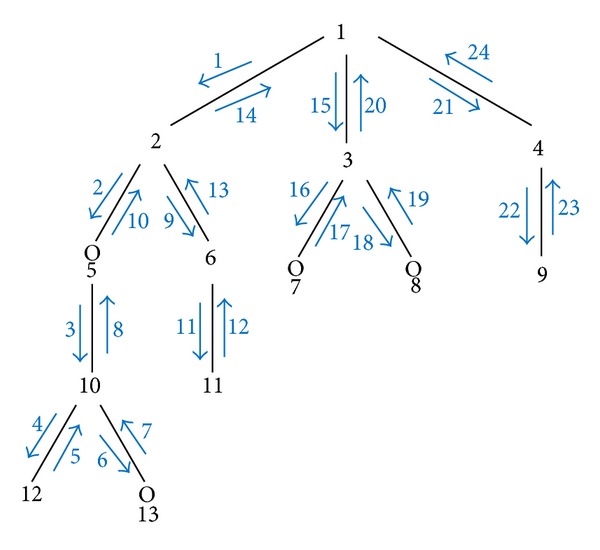
Process of generating the GBMD descriptor.

Generating the GBMD descriptor, the process of generating the shape descriptor (GBMD) for any molecule starts with head of the tree T of the graph (G). The atom name is represented in the descriptor as the grammar described below. Then, we move in a depth first algorithm to browse the tree. The bond type is represented before we visit and represent the next atom. For each breaking arc in the graph G (in case graph has cycle) we add a special character “=” to identify the cycles, as shown in Figure 4.

The same procedure is repeated until we visit again the head node/atom in the tree. Once the atom is visited again, the description of the outline shape of the molecule graph has been completed. Figure 4 shows the process of generating the descriptor (GBMD).

*Specification Rules*. The language used for writing the GBMD descriptor consists of series of characters and symbols. There are four generic encoding rules corresponding to the specification of atoms, bonds, ring closure, and disconnected parts. These rules are similar to the rules used in LWDOSM strings presented in [12]. The rules are described as follows.

**Rule 1 .**

Atoms are represented by their atomic symbols, usually two characters. The second character of the atomic symbol must be entered in lower case (e.g., “Br,” “C1,” “N,” and “O”).

**Rule 2 .**

The single, double, and triple bonds are represented by the symbols “–”, “=”, and “#”, respectively.

**Rule 3 .**

If the molecule graph is composed of more than one part (disconnected structures), the description of the disconnected compound is written as individual structures separated by “ . ” (period).

**Rule 4 .**

If the molecule graph is composed of rings (cycles), the special character “/” is added to each breaking arc to identify the rings.

The final GBDM for the aspirin molecule using the specification steps and the four rules described above is C–C–O–C–C–C–O–C–O–C–C–C–/C–C–C–C–O–C–O–C–C–CC–/C–C.

## 3. Intermolecular Similarity Calculation

The evaluation of the proposed descriptor will be measured by the performance of the similarity methods and will be compared to eight molecular descriptors which are ALOGP, MACCS, EPFP4, CDKFP, PCFP, SMILE, LWDOSM, and LINGO-DOSM. Two methods of similarity measures will be used. The first method will be used for evaluating the 2D fingerprint descriptors. In this method, the Tanimoto (TAN) coefficient was used which has been used for ligand based virtual screening for many years and is considered as a reference standard. The TAN was used for five types of descriptors (fingerprints) in this study (ALOGP, MACCS, EPFP4, CDKFP, PCFP, and LINGO-DOSM). The second similarity measure is the basic local alignment search tool (BLAST) string matching method which is used to evaluate the text-based molecular descriptors (SMILE, LWDOSM, and GBMD).

## 4. Experimental Design

In this section, we conduct experiments that show the usefulness of our proposed descriptor GBMD when used for similarity-based virtual screening. To evaluate the GBMD descriptor, GBMD was compared with different descriptors from Scitegic's Pipeline Pilot [15] and PaDEL-descriptor [16] software. These were canonical SMILES (SMILE) [17], 120-bit ALOGP, 166-bit MACCS, and 1024-bit Path fingerprints (EPFP4) from Scitegic's Pipeline Pilot and 1024-bit CDK (CDKFP) and 881-bit Pubchem fingerprints (PCFP) from the PaDEL-descriptor software.

Experiments were conducted over the most popular chemoinformatics database: the MDL drug data report (MDDR) [18] which has been used in our previous studies [12, 13, 19–21]. This database consists of 102,516 molecules. For the screening experiments, three data sets (DS1–DS3) were chosen from the MDDR database. The dataset DS1 contains 11 activity classes, with some of the classes involving actives that are structurally homogeneous and with others involving actives that are structurally heterogeneous (i.e., structurally diverse). The DS2 data set contains 10 homogeneous activity classes and the DS3 data set contains 10 heterogeneous activity classes. Details of these three data sets are listed in Tables 1, 2, and 3. Each row in the tables contains an activity class, the number of molecules belonging to the class, and the diversity of the class, which was computed as the mean pairwise Tanimoto similarity calculated across all pairs of molecules in the class using ECFP6.

The screening experiments were performed with 10 reference structures selected randomly from each activity class, which are the same references structures which were used in [12, 13, 19–21]. The recall results were averaged over each set of active molecules, where the recall is the percentage of the actives retrieved in the top-1% or the top-5% of the ranked list resulting from a similarity search.

## 5. Results and Discussion

The experiments are conducted to identify the possibility of using the GBMD descriptor in similarity-based virtual screening and then identifying the retrieval effectiveness of using such a descriptor. In this study, we compared the retrieval effectiveness of GBMD against six different descriptors using three different data sets, DS1–DS3. In addition, we compared the effectiveness of the new method with the LWDOSM and Lingo-DOSM [12, 13].

Selecting the best descriptors is based on their use in predicting the property/activity of a molecule from another molecule that is considered similar to it using a certain similarity method. For these descriptors, and for predicting the activity class of molecules, the best descriptors are these yielding the highest number of correct predictions (molecules with similar activity class), taking into account the total number of molecules having that activity class in the database used.

The results for the searches of DS1–DS3 are shown in Tables 4–9, respectively, using cutoffs of both 1% and 5%. Tables 4, 6, and 8 contain the results using the cutoff of 1%; and Tables 5, 7, and 9 contain the corresponding results using the cutoff of 5%. Each row in a table corresponds to one activity class and lists the recall for the top 1% and 5% of a sorted ranking when averaged over the ten searches for this activity class. The penultimate row in a table corresponds to the mean value for that descriptor when averaged over all of the activity classes for a data set. The descriptor with the best recall rate in each row is bolded. The bottom row in a table corresponds to the total number of bold cells for each descriptor type across the full set of activity classes.

Visual inspection of the recall values and the number of bold cells in Tables 4–9 enables comparisons to be made between the effectiveness of the GBMD descriptor and the various other descriptors. In addition, a more quantitative approach using the Kendall *W* test of concordance [22] was used to determine which descriptor performed best. This test was developed to quantify the level of agreement between multiple sets of rankings of the same set of objects, here and in previous works [12, 13, 19–21]. We used this approach to rank the effectiveness of different descriptor types. In the present context, the activity classes were considered as judges and the recall rates of the various descriptor types as objects. The outputs of the test are the value of the Kendall coefficient and the associated significance level, which indicates whether this value of the coefficient could have occurred by chance. If the value is significant (for which we used cutoff values of 0.01 or 0.05), then it is possible to give an overall ranking of the objects that have been ranked. The results of the Kendall analyses (for DS1–DS3) are reported in Table 10 and describe the top 1% and 5% ranking for the various descriptor types. In Table 10, the columns show the data set type, the value of the coefficient, the associated probability, and the ranking of the descriptor. The descriptors are ranked in decreasing order of screening effectiveness (if two descriptors have the same rank then they are ordered on the basis of the mean recall, that is, the mean values from the main tables of results).

We will use the DS1 results (in Tables 4 and 5) to illustrate the processing that took place. Here, it is shown that the GBMD descriptor has the best overall performance at the 1% and 5% cutoff. In addition, according to the total number of bold cells in Table 4, GBMD is the best performing descriptor across the 11 activity classes at the 1% and 5% cutoff. Table 10 shows that the value of the Kendall coefficient, 0.599, is significant at the 0.01 level of statistical significance; given that the result is significant, we can hence conclude that the overall ranking of the seven descriptors (for DS1 at 1% cutoff) is GBMD > CDKFP > SMILE > EPFP4 = MACCS > PCFP > ALOGP. The good performance of GBMD is not restricted to the top 1% for DS1, since it also gives the best results for the top-5% for DS1.

The results in Tables 6 and 7 show that the performance of GBMD is inferior to the best performance descriptors (CDKFP and EPFP4). The overall ranking of the 7 descriptors based on the Kendall coefficient in Table 10 (for DS2 top 1%) is CDKFP > EPFP4 > GBMD > SMILE > MACCS > ALOGP > PCFP. The results in Tables 6, 7, and 10 show that the GBMD descriptor performs least well when the active molecules being sought have a high degree of structural homogeneity. This is also the same in case of SMILE descriptor.

The best descriptor must be able to provide a scaffold-hopping capability for those cases where the actives belong to multiple structural classes. To determine to what degree GBMD descriptor obeys this principle, a GBMD descriptor was used to search for the most diverse set of active classes (DS3 data set). The search results for DS3 are shown in Tables 8 and 9. Here, the mean and total number of bold cells suggests that the GBMD descriptor is the best performing descriptor across the 10 activity classes at the 1% cutoff. GBMD also performs well and is comparable to standard descriptors (MACCS and EPFP) at the 5% cutoff.

Using the mean recall value as an evaluation criterion could be impartial to some descriptor type but not others and that is because some of the activity classes may contribute disproportionally to the overall value of mean recall. To avoid this bias, the effectiveness performance of different descriptors has been further investigated based on the total number of bold cells for each descriptor across the full set of activity classes, as shown in the bottom rows of Tables 4–9. These bold cell results are listed in Table 11. Visual inspection of the results in Table 11 shows clearly that the GBMD descriptor can provide a level of performance that is generally superior to the other descriptors. The only obvious exception is the DS2.

Retrieval results of top 1% and 5% of GBMD for data set DS1–DS3 compared with the LWDOSM and Lingo-DOSM are shown in Tables 12–14. The results show the performance improvements obtained by using GBMD compared to these two proposed descriptors for DS1. However, comparing GBMD with the LWDOSM and LINGO-DOSM descriptors in Table 13 for DS2 shows a lower performance because the LWDOSM and LINGO-DOSM are more sensitive on the shape of molecules, so they give better results for the low diversity dataset. Moreover, in Table 13, the retrieval results of top 1% and 5% for data set DS3 show that GBMD outperform the LWDOSM and Lingo-DOSM descriptors.

From the above results, it should be noted here that the main purpose of using several types of descriptor in the experiments was not a performance comparison only but also to show that our new descriptor GBMD is capable of representing and characterising the molecule structure and to show the possibility and feasibility of using GBMD for similarity-based virtual screening. However, the retrieval performance for any descriptor depends on the type of similarity methods used. Hence, we believe that using different text similarity searching methods with the GBDM descriptor such as the longest-common-subsequence (LCS) [23] and dynamic time warping (DTW) [24], will yield different results.

## 6. Conclusion

In this paper, we developed a new graph-based descriptor (GBMD) based on the canonical representations of molecules to provide a unique representation for each structure. Simulated virtual screening experiments with the MDL drug data report data sets compared the GBMD to many standard descriptors. The results show that the GBMD is working well for high diverse datasets, such that it outperformed all other descriptors for DS1 and DS3. Also, it outperformed the performance of LWDOSM and Lingo-DOSM descriptors that were proposed in our previous works.

## Figures and Tables

**Table 1:** MDDR activity classes for DS1 data set.

Activity index	Activity class	Active molecules	Pairwise similarity
31420	Renin inhibitors	1130	0.290
71523	HIV protease inhibitors	750	0.198
37110	Thrombin inhibitors	803	0.180
31432	Angiotensin II AT1 antagonists	943	0.229
42731	Substance P antagonists	1246	0.149
06233	5HT3 antagonist	752	0.140
06245	5HT reuptake inhibitors	359	0.122
07701	D2 antagonists	395	0.138
06235	5HT1A agonists	827	0.133
78374	Protein kinase C inhibitors	453	0.120
78331	Cyclooxygenase inhibitors	636	0.108

**Table 2:** MDDR activity classes for DS2 data set.

Activity index	Activity class	Active molecules	Pairwise similarity
07707	Adenosine (A1) agonists	207	0.229
07708	Adenosine (A2) agonists	156	0.305
31420	Renin inhibitors	1130	0.290
42710	CCK agonists	111	0.361
64100	Monocyclic lactams	1346	0.336
64200	Cephalosporins	113	0.322
64220	Carbacephems	1051	0.269
64500	Carbapenems	126	0.260
64350	Tribactams	388	0.305
75755	Vitamin D analogues	455	0.386

**Table 3:** MDDR activity classes for DS3 data set.

Activity index	Activity class	Active molecules	Pairwise similarity
09249	Muscarinic (M1) agonists	900	0.111
12455	NMDA receptor antagonists	1400	0.098
12464	Nitric oxide synthase inhibitors	505	0.102
31281	Dopamine hydroxylase inhibitors	106	0.125
43210	Aldose reductase inhibitors	957	0.119
71522	Reverse transcriptase inhibitors	700	0.103
75721	Aromatase inhibitors	636	0.110
78331	Cyclooxygenase inhibitors	636	0.108
78348	Phospholipase A2 inhibitors	617	0.123
78351	Lipoxygenase inhibitors	2111	0.113

**Table 4:** Retrieval results of top 1% for data set DS1.

Activity index	GBMD	SMILE	PCFP	ALOGP	MACCS	EPFP4	CDKFP
31420	**60.93**	53.69	26.13	22.06	28.65	34.75	41.8
71523	26.49	**26.84**	9.61	13.72	14.71	14.29	19.6
37110	**20.84**	15.52	12.38	9.26	17.99	18.8	18.74
31432	**40.71**	34.96	15.55	16.52	24.52	22.81	25.75
42731	**16.86**	16.20	9.63	6.05	8.18	10.08	12.27
6233	8.16	7.30	6.8	7.98	8.8	8.35	**9.47**
6245	5.75	5.47	4.11	3.66	4.94	5.61	**7.21**
7701	**10.86**	7.79	4.62	5.86	7.39	6.75	7.77
6235	7.83	7.43	4.27	6.22	6.91	6.55	**8.29**
78374	8.03	7.92	**13.16**	7.81	6.02	8.01	10.64
78331	6.09	5.98	5.13	4.11	**6.33**	4.94	5.72

Mean	**19.32**	17.19	10.13	9.39	12.22	12.81	15.21

Bold cells	6	1	1	0	1	0	3

**Table 5:** Retrieval results of top 5% for data set DS1.

Activity index	GBMD	SMILE	PCFP	ALOGP	MACCS	EPFP4	CDKFP
31420	82.74	**83.45**	45.95	45.08	55.41	76.76	80.27
71523	52.92	**57.04**	19.73	33.38	29.97	33.31	37.92
37110	31.47	33.77	27.99	26.71	34.7	**39.96**	37.26
31432	**74.24**	66.27	33.73	39.37	48.29	41.01	51.46
42731	**28.84**	24.00	19.32	12.91	19.36	20.71	23.2
6233	18.28	14.94	17	20.47	**24.07**	20	19.92
6245	13.69	13.46	10.08	10.59	11.06	12.65	**17.88**
7701	**24.61**	19.92	11.62	13.6	22.34	17.69	18.86
6235	19.35	**21.04**	13.51	14.71	20.33	17.82	19.21
78374	15.8	13.05	**18.1**	14.71	11.73	12.59	15.11
78331	13.51	14.25	11.23	9.97	**14.35**	9.37	10.55

Mean	**34.13**	32.84	20.75	21.95	26.51	27.44	30.15

Bold cells	4	2	1	0	2	1	1

**Table 6:** Retrieval results of top 1% for data set DS2.

Activity index	GBMD	SMILE	PCFP	ALOGP	MACCS	EPFP4	CDKFP
7707	60.39	60.87	43.69	64.56	63.35	72.52	**72.82**
7708	68.65	82.90	78	91.03	71.03	97.94	**100**
31420	64.59	**68.56**	39.05	37.91	29.61	43.14	49.2
64100	74.27	79.09	46.36	79.27	86.45	**86.64**	83.55
64200	83.2	67.13	69.68	81.59	83.72	**91.14**	91.03
64220	49.04	39.02	47.86	47.41	53.48	66.16	**82.68**
64500	59.66	65.69	46.26	30.94	52.99	**78.49**	67.9
64300	76.48	30.64	22.72	34.64	52.88	**87.36**	85.68
65000	**80.18**	62.14	45.97	65.06	57.88	75.45	70.34
75755	93.72	93.61	86.26	85.62	66.94	92.38	**96.45**

Mean	71.01	64.97	52.59	61.8	61.83	79.12	**79.97**

Bold cells	1	1	0	0	1	4	5

**Table 7:** Retrieval results of top 5% for data set DS2.

Activity index	GBMD	SMILE	PCFP	ALOGP	MACCS	EPFP4	CDKFP
7707	71.12	71.41	71.6	75.49	**78.2**	77.96	75.39
7708	83.42	98.26	90.32	99.74	97.23	**100**	**100**
31420	90.5	**93.05**	55.58	61.15	55.9	81.73	88.81
64100	87.45	89.64	81.64	85.82	**98.45**	95.64	94.55
64200	96.53	88.24	89.6	95.36	97.87	**99.93**	99.63
64220	83.63	72.77	67.41	65.27	78.39	**99.91**	99.82
64500	76.84	93.51	78.89	59.66	85.77	**99.84**	97.41
64300	90.32	47.92	40.64	56.24	83.12	**99.84**	99.6
65000	**96.12**	71.01	71.91	85.5	81.34	86.59	85.43
75755	97.09	97.53	96.81	98.04	86.96	97.8	**98.13**

Mean	87.30	82.33	74.44	78.23	84.32	**93.92**	93.88

Bold cells	1	1	0	0	2	6	2

**Table 8:** Retrieval results of top 1% for data set DS3.

Activity index	GBMD	SMILE	PCFP	ALOGP	MACCS	EPFP4	CDKFP
9249	14.64	**16.00**	10.11	11.71	11.45	15.44	13.6
12455	5.81	**7.18**	6.81	3.94	4.94	6.27	6.23
12464	3.17	9.40	11.13	9.31	**19.62**	8.65	11.61
31281	16.38	16.19	12.19	**22.29**	21.05	17.33	16.48
43210	9.58	9.16	4.6	5.84	**10.06**	6.98	7.04
71522	**7.56**	4.36	6.27	3.71	5.38	6.62	5.75
75721	**26.91**	19.54	22.09	17.8	19.39	22.24	22.33
78331	**6.83**	5.81	4.16	3.97	6.08	4.55	5.09
78348	**12.73**	4.70	3.58	4.24	3.27	4.29	3.69
78351	11.05	14.46	12.69	8.32	13.32	15.01	**15.49**

Mean	**11.47**	10.68	9.36	9.11	**11.46**	10.74	10.73

Bold cells	5	2	0	1	2	0	1

**Table 9:** Retrieval results of top 5% for data set DS3.

Activity index	GBMD	SMILE	PCFP	ALOGP	MACCS	EPFP4	CDKFP
9249	24.19	28.04	16.23	20.51	21.97	**29.63**	21.46
12455	10.21	**12.55**	11.87	8.03	9.26	8.51	9.39
12464	7.2	18.69	22.96	19.8	**39.01**	18.19	21.69
31281	25.62	27.52	23.9	33.24	**40.19**	34.95	27.71
43210	10.4	**20.38**	11.42	14.05	18.61	12.63	13.19
71522	**14.1**	9.17	12.86	8.5	12.02	13.89	10.92
75721	**35.72**	27.18	29.72	29.15	27.86	30.79	30.76
78331	**14.38**	12.25	9.61	9.26	13.75	10.17	9.43
78348	**27.14**	9.33	7.43	9.72	8.6	10.47	9.01
78351	12.93	16.61	15.03	10.58	12.89	**16.7**	16.16

Mean	18.19	18.17	16.1	16.28	**20.42**	18.59	16.97

Bold cells	4	2	0	0	3	2	0

**Table 10:** Rankings of various types of descriptors based on Kendall *W* test results: Top 1 and 5%.

Data set	Recall type	*W*	*P*	Ranking
DS1	Top 1%	0.599	<0.01	GBMD > CDKFP > SMILE > EPFP4 = MACCS > PCFP > ALOGP
	Top 5%	0.372	<0.01	GBMD > SMILE > CDKFP > MACCS > EPFP4> ALOGP > PCFP

DS2	Top 1%	0.503	<0.01	CDKFP > EPFP4 > GBMD > SMILE > MACCS > ALOGP > PCFP
	Top 5%	0.443	<0.01	EPFP4 > CDKFP > MACCS > GBMD > ALOGP > SMILE > PCFP

DS3	Top 1%	0.189	<0.01	GBMD > EPFP4 = CDKFP = SMILE > MACCS > PCFP > ALOGP
	Top 5%	0.141	<0.01	EPFP4 > GBMD > MACCS > CDKFP > PCFP > SMILE > ALOGP

**Table 11:** Numbers of bold cells for mean recall of actives using different descriptors: DS1-DS3 Top 1% and 5%.

Data set	GBMD	SMILE	PCFP	ALOGP	MACCS	EPFP4	CDKFP
	Top 1%						
DS1	6	1	1	0	1	0	3
DS2	1	1	0	0	1	4	5
DS3	5	2	0	1	2	0	1

	Top 5%						
DS1	4	2	1	0	2	1	1
DS2	1	1	0	0	2	6	2
DS3	4	2	0	0	3	2	0

**Table 12:** Retrieval results of top 1% and 5% for data set DS1 compared with LWDOSM and Lingo-DOSM.

Activity index	Top 1%			Top 5%		
	GBMD	LINGO-DOSM	LWDOSM	GBMD	LINGO-DOSM	LWDOSM
31420	60.93	61.1	**73.21**	82.74	84.82	**94.23**
71523	**26.49**	26.1	20.4	**52.92**	50.11	43.5
37110	**20.84**	17.37	12.18	**31.47**	28.19	23.8
31432	**40.71**	38.63	36.03	74.24	**75.27**	68.18
42731	**16.86**	11.86	14.34	**28.84**	21.62	27.51
6233	8.16	**11.46**	9.36	18.28	**25.73**	16.32
6245	5.75	4.66	**5.98**	13.69	10.92	**14.92**
7701	**10.86**	10.38	8.98	**24.61**	22.99	24.31
6235	7.83	**10.34**	8.23	19.35	**26.43**	21.42
78374	8.03	**12.01**	11.66	15.8	18.3	**20.4**
78331	**6.09**	5.8	4.79	**13.51**	10.16	12.98

Mean	**19.32**	19.06	18.65	**34.13**	34.04	33.42

Bold cells	7	3	2	6	3	3

**Table 13:** Retrieval results of top 1% and 5% for data set DS2 compared with LWDOSM and Lingo-DOSM.

Activity index	Top 1%			Top 5%		
	GBMD	LINGO-DOSM	LWDOSM	GBMD	LINGO-DOSM	LWDOSM
7707	**60.39**	60.29	54.71	71.12	**71.21**	70.34
7708	68.65	**86.77**	82	83.42	**97.87**	93.29
31420	64.59	66.77	**66.91**	90.5	84.81	**93.18**
64100	74.27	79	**93.27**	87.45	98.45	**98.73**
64200	83.2	**86.16**	86.07	96.53	99.55	**99.77**
64220	49.04	**71.4**	68.6	83.63	**98.85**	92.23
64500	59.66	**76.66**	74.65	76.84	**99.77**	95.56
64300	76.48	**92.65**	73.52	90.32	**100**	91.28
65000	**80.18**	51.19	51.06	**96.12**	78.24	67.8
75755	93.72	**97.89**	96.3	97.09	**98.06**	97.91

Mean	71.01	**76.86**	74.71	87.3	**92.68**	90

Bold cells	2	7	2	1	6	3

**Table 14:** Retrieval results of top 1% and 5% for data set DS3 compared with LWDOSM and Lingo-DOSM.

Activity index	Top 1%			Top 5%		
	GBMD	LINGO-DOSM	LWDOSM	GBMD	LINGO-DOSM	LWDOSM
9249	14.64	**15.48**	9.84	24.19	**26.9**	16.24
12455	5.81	**9.81**	8.29	10.21	14.62	**14.86**
12464	3.17	**5.32**	4.8	7.2	11.27	12.1
31281	**16.38**	16.19	12.1	25.62	**27.33**	21.9
43210	5.58	4.56	**6.07**	10.4	10.4	**15.19**
71522	**3.56**	2.36	2.98	**7.1**	5.89	6.34
75721	**26.91**	22.96	23.67	**35.72**	28.17	33.4
78331	6.83	**9.57**	7.59	14.38	15.48	**16.88**
78348	**12.73**	7.09	7.69	**27.14**	13.9	18.05
78351	**11.05**	10.31	8.24	12.93	**12.97**	11.46

Mean	**10.66**	10.36	9.13	**17.48**	16.69	16.64

Bold cells	6	4	1	4	3	3

## References

[1] Christie BD, Leland BA, Nourse JG (1993). Structure searching in chemical databases by direct lookup methods. *Journal of Chemical Information and Computer Sciences*.

[2] Fisanick W, Lipkus AH, Rusinko A (1994). Similarity searching on CAS registry substances. 2. 2D structural similarity. *Journal of Chemical Information and Computer Sciences*.

[3] Figueras J (1972). Substructure search by set reduction. *Journal of Chemical Documentation*.

[4] Sussenguth EH (1965). A graph-theoretic algorithm for matching chemical structures. *Journal of Chemical Documentation*.

[5] Willett P, Barnard JM, Downs GM (1998). Chemical similarity searching. *Journal of Chemical Information and Computer Sciences*.

[6] Johnson MA, Maggiora GM (1990). *Concepts and Application of Molecular Similarity*.

[7] Maldonado AG, Doucet JP, Petitjean M, Fan B (2006). Molecular similarity and diversity in chemoinformatics: from theory to applications. *Molecular Diversity*.

[8] Rarey M, Dixon JS (1998). Feature trees: a new molecular similarity measure based on tree matching. *Journal of Computer-Aided Molecular Design*.

[9] Rarey M, Stahl M (2001). Similarity searching in large combinatorial chemistry spaces. *Journal of Computer-Aided Molecular Design*.

[10] Garey MR, Johnson DS (1977). The rectilinear Steiner tree problem is NP-complete. *SIAM Journal on Applied Mathematics*.

[11] Rush TS, Grant JA, Mosyak L, Nicholls A (2005). A shape-based 3-D scaffold hopping method and its application to a bacterial protein-protein interaction. *Journal of Medicinal Chemistry*.

[12] Hentabli H, Salim N, Abdo A, Saeed F (2012). LWDOSM: language for writing descriptors of outline shape of molecules. *Advanced Machine Learning Technologies and Applications*.

[13] Hentabli H, Salim N, Abdo A, Saeed F (2013). LINGO-DOSM: LINGO for descriptors of outline shape of molecules. *Intelligent Information and Database Systems*.

[14] Morgan HL (1965). The generation of a unique machine description for chemical structures—a technique developed at chemical abstracts service. *Journal of Chemical Documentation*.

[15] (2008). *Pipeline Pilot*.

[16] Yap CW (2011). PaDEL-descriptor: an open source software to calculate molecular descriptors and fingerprints. *Journal of Computational Chemistry*.

[17] Weininger D (1988). SMILES, a chemical language and information system. 1. Introduction to methodology and encoding rules. *Journal of Chemical Information and Computer Sciences*.

[18] http://accelrys.com/.

[19] Abdo A, Chen B, Mueller C, Salim N, Willett P (2010). Ligand-based virtual screening using bayesian networks. *Journal of Chemical Information and Modeling*.

[20] Abdo A, Salim N (2011). New fragment weighting scheme for the Bayesian inference network in ligand-based virtual screening. *Journal of Chemical Information and Modeling*.

[21] Abdo A, Salim N, Ahmed A (2011). Implementing relevance feedback in ligand-based virtual screening using Bayesian inference network. *Journal of Biomolecular Screening*.

[22] Siegel S, Castellan NJ (1988). *Nonparametric Statistics for the BehaVioral Sciences*.

[23] Bergroth L, Hakonen H, Raita T A survey of longest common subsequence algorithms.

[24] Müller M Dynamic time warping.

